# Editorial: *Mycoplasma pneumoniae* Clinical Manifestations, Microbiology, and Immunology

**DOI:** 10.3389/fmicb.2017.01916

**Published:** 2017-09-29

**Authors:** Ran Nir-Paz, Takeshi Saraya, Takashi Shimizu, Annemarie Van Rossum, Cécile Bébéar

**Affiliations:** ^1^Department of Clinical Microbiology and Infectious Diseases, Hadassah Medical Center, Jerusalem, Israel; ^2^Department of Respiratory Medicine, Kyorin University, Mitaka, Japan; ^3^Yamaguchi University, Yamaguchi, Japan; ^4^Sophia Children's Hospital, Rotterdam, Netherlands; ^5^Mycoplasmal and Chlamydial Infections in Humans, University of Bordeaux - INRA, USC EA 3671, Bordeaux, France

**Keywords:** *Mycoplasma pneumoniae*, mycoplasma infections, epidemiology, pneumonia, respiratory tract infections

*Mycoplasma pneumoniae* is a major human pathogen that causes both upper and lower respiratory infections, and is one of the leading causes of community-acquired pneumonia (CAP), accounting for 11–15% of CAP throughout the world (Brown et al.; Kishaba; Meyer Sauteur et al.; Saraya). *M. pneumoniae* is also one of the smallest free-living human pathogens, with a genome size of less than 700 genes (Spuesens et al.). In this volume of Frontiers we have explored few of many aspects related to this major human pathogen. The purpose of this topic was to thoroughly review and to create a body of knowledge encompassing both medical and biological information gathered in both the western world and Asia.

Saraya contributed a nice historical perspective on the discovery of *M. pneumoniae* and a few authors contributed an updated and fresh look on the clinical manifestations of *M. pneumoniae* (Kishaba; Meyer Sauteur et al.; Izumikawa; Narita; Tanaka; Parrott et al.). The unique epidemiological challenges presented by this pathogen include both outbreaks and the “Olympic” (3–5 years) intervals between epidemics, which reported here in both Japan and the UK (Brown et al.; Parrott et al.; Yamazaki and Kenri). Since its discovery, the main challenge for diagnostic labs and clinicians was its fastidious growth that led to a reliance on serology, and later on, amplification techniques for diagnosis. Two reviews cover diagnostics here (Diaz and Winchell, Loens and Ieven). An additional review combines the antibody response to epidemiological aspects as a means to explain the epidemiology (Dumke and Jacobs). Pathogenesis is covered by 2 topics: One on the bacterial aspect and the other on the host inflammatory response to *M. pneumoniae* infection (Miyata and Hamaguchi; Shimizu). Additionally, Spuesens et al. explored the possible role of genomics in elucidating the differentiation between sick and carrier patients. Obviously, for each infectious agent, treatment and cure is the goal, if prevention is not possible (yet). However, since *M. pneumoniae* is lacking a cell wall, antibiotic choices are limited to the use of macrolides and related antibiotics, tetracyclines and quinolones, and some of those classes have limited use in the pediatric populations (Meyer Sauteur et al.; Pereyre et al.). Pereyre et al. summarized the emerging macrolide resistance and Balish and Distelhorst explored the possible use of new agents in the future.

Obviously, not everything can be covered in depth and many of the topics explored here raise many new questions that remains to be answered, by the scientific community.

## Author contributions

RN wrote the first draft; TSa, TSh, AV, and CB reviewed commented and edited the manuscript. All authors have approved the manuscript.

### Conflict of interest statement

The authors declare that the research was conducted in the absence of any commercial or financial relationships that could be construed as a potential conflict of interest.

